# Complete Genome Sequence of an Uncultivated Freshwater *Flavobacterium* sp. Strain

**DOI:** 10.1128/mra.00659-22

**Published:** 2022-09-27

**Authors:** Julia Bernet, Lucas Serra Moncadas, Adrian-Stefan Andrei

**Affiliations:** a Department of Biology, Swiss Federal Institute of Technology in Zurich, Zurich, Switzerland; b Microbial Evogenomics Lab, Limnological Station, Department of Plant and Microbial Biology, University of Zurich, Kilchberg, Switzerland; University of Delaware

## Abstract

Here, we report the complete metagenome-assembled genome of an uncultivated freshwater *Flavobacterium* sp. recovered from a nonaxenic Amoebozoa sp. culture. The chromosome was obtained from a metagenomic long-read sequencing run and was assembled as a circular element at a 51× coverage, length of 3.8 Mbp and a G+C content of 37.37%.

## ANNOUNCEMENT

Flavobacterium genus exhibits a cosmopolitan distribution encompassing 368 species thriving from sea ice and saline lakes to fish flesh (1). Despite the large taxonomic and physiologic radiation of the genus, a deeper understanding of the factors that shape the niche partitioning in large species pools is hampered by our inability to determine the nutritional requirements for abundant microbial populations and the lack of high-quality genomes.

An Amoebozoa sp. was enriched from the perialpine Lake Zurich (47°18’N, 8°34’E, Switzerland). The organism was maintained in tissue culture flasks filled with 50 mL of autoclaved Swiss Aplina (Pearlwater, Termen, Switzerland) water and fed with cyanobacterium Planktothrix rubescens strain A7. Three nonaxenic stationary-phase cultures were filtered through 5 and 0.2-μm-pore-size polycarbonate filters to concentrate the microbial biomass. The 0.2 μm filters were cut into small pieces and subjected to DNA extraction using the MagAttract HMW DNA kit (Qiagen, Hilden, Germany). The obtained DNA was further purified with Beckman Coulter AMPure XP magnetic beads and pooled prior to metagenomic sequencing on a Nanopore PromethION platform using a FLO-PRO002 (R9.4.1) flow cell. The sequencing 1D library was constructed with the SQK-LSK110 Ligation Sequencing kit (ONT, Oxford, UK) in conformity with manufacturer's instructions without any prior DNA fragmentation or size selection.

Obtained raw reads (approx. 2 million reads; mean read length 9 kbp, median read quality 11.7, *N*_50_ 14.4 kbp) were basecalled and quality trimmed (Q score 7) with Guppy 5.1.15 prior to assembly with Flye 2.9-b1768 (2). The recovered circular chromosome (3 808 583 bp) was classified using GTDB-Tk v1.4.0 toolkit (3) and by comparing its 16S rRNA gene (predicted with barrnap 0.9) against SILVA database (version 138) (4). Potential genome contamination was assessed by using CheckM v1.1.3 (5) (97% complete, 0.65% contamination). Coding DNA sequences and tRNAs were predicted by Prokka 1.12 (6) and NCBI’s PGAP pipeline. BlastKOALA (7) was used to assign KO identifiers to orthologous genes. Inferences of complete metabolic pathways and general biological functions were conducted with the online KEGG mapping tools using summarized KO numbers. PFAM domains were identified in the proteome using the script pfam_scan.pl with PFAM database release 32 (8). Growth rate was predicted with the R package gRodon (9), while anti-phage systems were predicted with DefenseFinder (10). Default parameters were used except where otherwise specified.

The metagenome-assembled genome (strain JAD_PAG50586_2) was classified as belonging to *Flavobacterium* genus by both GTDB and SILVA (96.6% similarity to closest Flavobacterium buctense) databases. It was found to encode 3 821 CDS, 42 tRNAs and to possess 3 rRNA operons. Genome-inferred metabolic reconstructions depicted a copiotroph diderm bacterium with a high growth rate and aerobic heterotrophic lifestyle. Predicted amino acid transporters likely alleviate potential auxotrophies (for 6 proteinogenic amino acids) and can contribute (through degradation pathways; l-Leucine) together with mannose degradation-Embden-Meyerhof glycolysis (coupled through α-d-Glucose-1P) to replenishing the acetyl-CoA pool needed to fuel the TCA cycle.

### Data availability.

All sequence data are available through the National Center for Biotechnology Information (NCBI) via the BioProject PRJNA824509 (CP096136.1, GCA_023213215.1, SRX14779367). Additional proteome annotations (KEGG, Prokka, and Pfam) are available in figshare repository: 10.6084/m9.figshare.20160587.
